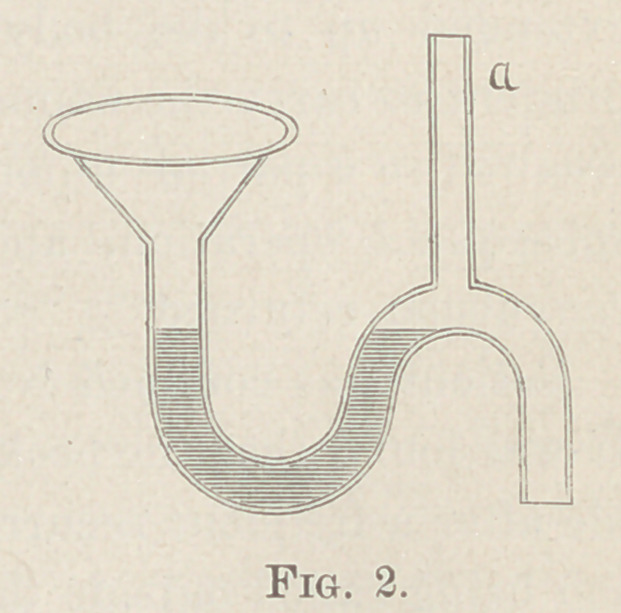# Chicago Medical Society

**Published:** 1881-04

**Authors:** 


### ARTICLE VII.

CHICAGO MEDICAL SOCIETY. Stated meeting March 7, 1881.

Dr. R. G. Bogue, President, in the chair. Drs. Henry P. Newman and Charles Krusemarck were elected members. Dr. R. Tilley exhibited the *Norton Attachment* to waste-water pipes. It presents the advantage of shutting, by means of a faucet, any communication between the waste-water pipe and the house. Dr. Tilley then demonstrated the worthlessness of common S traps, in which most of the water is syphoned off, as in Figure 1, the dark lines representing all that is left of the water, which soon evaporates and leaves a free entrance for the sewer gas.

FIG. 1.

FIG. 2.

Figure 2 shows a contrivance of Dr. Tilley which allows a large quantity of water to remain in the S trap by means of atmospheric pressure through a communication with the outside air by the pipe *a*, which should be carried up to the roof of the house, and would thus ventilate the waste-water pipe. This arrangement had the preference of several members present, but, as Dr. Tilley remarked, it is best adapted to new buildings, and its introduction in old ones is somewhat expensive.

## Figures and Tables

**Fig. 1. f1:**
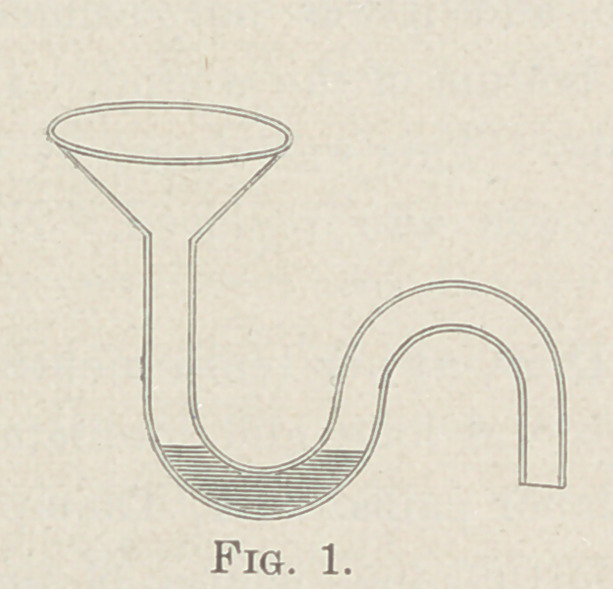


**Fig. 2. f2:**